# Exercise patterns in older adults instructed to follow moderate- or high-intensity exercise protocol – the generation 100 study

**DOI:** 10.1186/s12877-018-0900-6

**Published:** 2018-09-10

**Authors:** Line Skarsem Reitlo, Silvana Bucher Sandbakk, Hallgeir Viken, Nils Petter Aspvik, Jan Erik Ingebrigtsen, Xiangchun Tan, Ulrik Wisløff, Dorthe Stensvold

**Affiliations:** 1 K.G. Jebsen Center of Exercise in Medicine at Department of Circulation and Medical Imaging, Faculty of Medicine and Health Sciences, Norwegian University of Science and Technology, Trondheim, Norway; 2 Department of Cardiology, St Olavs Hospital, Trondheim University Hospital, Trondheim, Norway; 3 Norwegian National Advisory Unit on Exercise Training as Medicine for Cardiopulmonary Conditions, St. Olav’s Hospital, Trondheim, Norway; 4 Department of Sociology and Political Science, Faculty of Social and Educational Sciences, Norwegian University of Science and Technology, Trondheim, Norway; 5 Department of Neuroscience and Movement Science, Faculty of Medicine and Health Sciences, Norwegian University of Science and Technology, Trondheim, Norway; 6 School of Human Movement & Nutrition Sciences, University of Queensland, Brisbane, Australia

**Keywords:** Aging, Aged, Exercise, High-intensity interval training

## Abstract

**Background:**

Making older adults exercise and keeping them in exercise programs is a major challenge. Understanding how older adults prefer to exercise may help developing tailored exercise programs and increase sustained exercise participation in ageing populations. We aimed to describe exercise patterns, including frequency, intensity, type, location and social setting of exercise, in older adults instructed to follow continuous moderate-intensity training (MCT) or high-intensity interval training (HIIT) over a one-year period.

**Methods:**

Frequency, intensity, type, location and social setting (alone vs. together with others) of exercise were assessed using exercise logs from 618 older adults (aged 70–77 years) randomized to MCT or HIIT. All participants completed exercise logs after each exercise session they performed during one year. Pearson Chi-square tests were run to assess the association between intensity, type, location and social setting of exercise with training group.

**Results:**

Both groups performed 2.2 ± 1.3 exercise sessions per week during the year. Walking was the most common exercise type in both groups, but MCT had a higher proportion of walking sessions than HIIT (54.2% vs. 41.1%, *p* < 0.01). Compared to MCT, HIIT had a higher proportion of sessions with cycling (14.2% vs. 9.8%, *p* < 0.01), combined endurance and resistance training (10.3% vs. 7.5%, *p* < 0.01), jogging (6.5% vs. 3.2%, *p* < 0.01) and swimming (2.6% vs. 1.7%, *p* < 0.01). Outdoors was the most common exercise location in both training groups (67.8 and 59.1% of all sessions in MCT and HIIT, respectively). Compared to MCT, HIIT had a higher proportion of sessions at a gym (21.4% vs. 17.5%, *p* < 0.01) and sports facility (9.8% vs. 7.6%, *p* < 0.01). Both groups performed an equal amount of sessions alone and together with others, but women had a higher proportion of sessions together with others compared to men (56% vs. 44%, *p* < 0.01).

**Conclusion:**

This is the first study that has followed older adults instructed to perform MCT or HIIT over a one-year period, collected data from each exercise session they performed and provided important knowledge about their exercise patterns. This novel information may help researchers and clinicians to develop tailored exercise programs in an ageing population.

## Background

The world population is ageing and the number of older adults with chronic health conditions and physical limitations is expected to increase. This, in turn, could lead to an increased burden on healthcare services [1]. Regular physical activity is an important component of successful ageing and reduces the risk of developing several age- and lifestyle related diseases such as cardiovascular disease, dementia and type 2 diabetes [2–7]. However, making older adults exercise and keeping them in exercise programs is a major challenge [8]. Understanding how older adults prefer to exercise may help developing tailored exercise programs and increase sustained exercise participation in ageing populations.

Many exercise interventions have been conducted under controlled laboratory conditions [9], but we do not know how older adults prefer to exercise when they are not under controlled settings and are free to choose type, location and social setting (e.g. alone vs. together with others) of exercise. Furthermore, it has been shown that high-intensity interval training (HIIT) can induce superior changes in health-related markers compared to continuous moderate-intensity training (MCT) [10–13], also in older adults [14, 15]. The scientific interest in HIIT has greatly increased during recent years [9], but larger and longer studies under free-living conditions are needed to investigate whether HIIT is feasible as a public health strategy among older adults [9, 16]. Therefore, detailed information about older adults exercise patterns with MCT versus HIIT outside laboratory conditions is of particular interest.

Furthermore, exercise initiatives should include strategies that will appeal to various subgroups of older adults. Disparities in physical activity levels between older women and men exist [17, 18], and sex differences are therefore an important consideration when examining exercise patterns.

The aim of this study was to describe exercise patterns, including frequency, intensity, type, location and social setting of exercise, in older adults instructed to follow MCT or HIIT over a one-year period. We also aimed to describe sex differences in exercise patterns in older adults.

## Methods

### Study participants

Between August 2012 and June 2013, all men and women born between years 1936 to 1942 (aged 70–77 years), with a permanent address in the municipality of Trondheim, Norway, were invited to participate in a randomized controlled trial, the Generation 100 study. The primary aim of Generation 100 is to determine the effect of five years of exercise training on morbidity and mortality. The Generation 100 study protocol and study sample characteristics have been published previously [19].

**Fig. 1:**
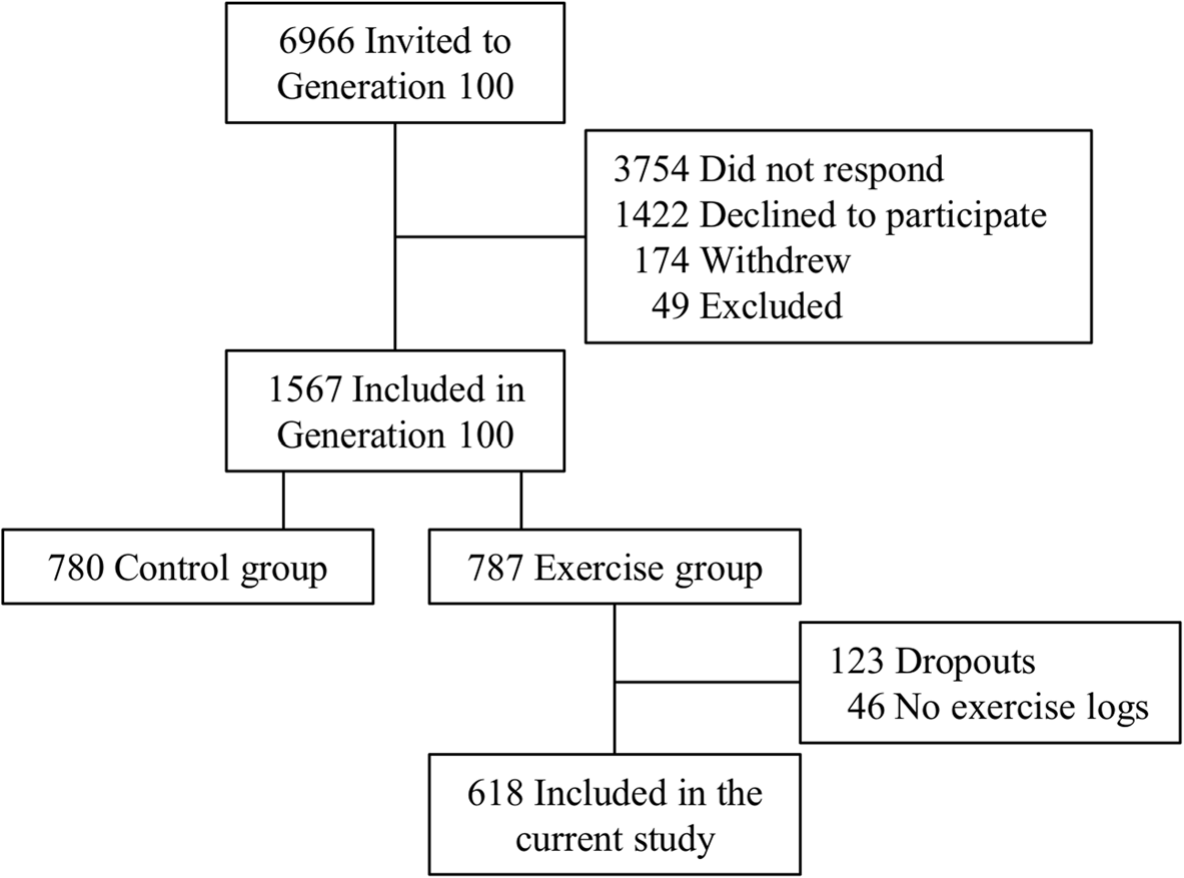
Study flowchart

In total, 1567 participants (790 women) met the inclusion criteria, fulfilled baseline testing and were randomized 1:1 into an exercise training group or to a control group. The exercise training group was further randomized 1:1 to either MCT or HIIT. Participants in the exercise groups were instructed to fill in exercise logs after each exercise session they performed. Data in the present study is based on the exercise logs from the first year of the intervention. Therefore, only participants in the exercise groups were included in the present study (*n* = 787). Dropouts in the exercise groups during the first year (*n* = 123) and those with no exercise logs (*n* = 46) were excluded. A total of 618 participants (291 women) were included in the analyses (Fig. 1). The study was approved by the Regional Committee for Medical Research Ethics (REK sør-øst B: 2015/945) and all participants gave their written informed consent before participation.

### Exercise intervention

The MCT group was prescribed two weekly exercise sessions of 50-min continuous activity at 70% of peak heart rate, or approximately 13 on the Borg 6–20 rating of perceived exertion (RPE) scale [20]. The HIIT group was prescribed two exercise sessions a week with 10-min warm-up followed by 4 × 4 min intervals at 85–95% of peak heart rate, or approximately 16 on the Borg 6–20 RPE scale. The participants were given individual oral and written information about the training methods, including information about frequency, duration, intensity and examples of exercise sessions. The participants were free to exercise individually, with an exercise type and at a location of their own choosing. Every sixth week the participants met for a supervised spinning session where they exercised with a heart rate monitor. These exercise sessions gave the participants an opportunity to control their intensity during exercise. In addition, organized group exercise was offered twice per week for motivational purposes. Attendance to these exercises was voluntary and the activity performed varied between indoor and outdoor activities such as walking, jogging and aerobics [19]. Besides the two prescribed exercise sessions, the participants were free to exercise as desired.

### Assessment of exercise patterns

Exercise was defined as planned, structured activities, for instance going for walks, skiing, swimming and doing sports, but also as unplanned activities that the participants experienced as exercise. The participants were asked to fill in exercise logs immediately after each exercise session they performed throughout the year and send them to the research center either in prepaid envelopes monthly, or to use internet-based forms following each exercise session [21]. Exercise frequency was calculated as the mean number of sessions reported per week during the year. To assess intensity of exercise the participants reported their subjective RPE on a Borg scale ranging from 6 to 20 [20]. The participants were asked to report the mean intensity level during the exercise session. Ratings from 6 to 10 were classified as low intensity, 11 to 14 as moderate intensity, and 15 to 20 as high intensity. Duration of exercise was measured with a 4-point scale: less than 15 min, 15–29 min, 30 min to 1 h, and more than 1 h. Less than 15 min and 15–29 min was combined due to a low response rate on these response options (1.1 and 8.7% of the total number of exercise sessions, respectively).

To measure exercise type the participants were instructed to choose from the following response options: walking, jogging, cycling, dancing, cross-country skiing, swimming, golf, resistance training and an open-ended response option. Answers in the open-ended response option were categorized into: combined endurance and resistance training, other type of endurance training (e.g. treadmill, aerobic), domestic activities (e.g. housework, gardening), and other (e.g. bowling, horseback riding). Golf was categorized as “other” due to a low response rate (0.5% of the total number of exercise sessions).

The question used to assess location of exercise had the following response options: home, outdoor in nearby area, nature, gym, indoor- and outdoor sports facility. Indoor- and outdoor sports facility was categorized as “sports facility” due to a low response rate on the outdoor sports facility option (1%). For social setting of exercise, the response options were: exercised alone, exercised together with others, and organized by Generation 100.

### Demographics and health characteristics

The baseline testing included clinical examinations, physical tests and questionnaires about health and lifestyle. Age and sex were obtained from the National Population Registry. A previously described questionnaire provided information on physical activity level and sedentary time at baseline [19]. Detailed protocol for assessment of body weight (kg), body height (cm) and body mass index (BMI; kg/m^2^) is described elsewhere [19]. Testing of peak oxygen uptake (VO_2peak_^;^ mL/kg/min) was performed either as walking on a treadmill or cycling on a stationary bike. The test started with 10 min at a chosen warm-up speed. Approximately every two minutes, either the incline of the treadmill was increased by 2%, or the speed was increased by 1 km/h. The test protocol ended when participants were no longer able to carry a workload due to exhaustion or until all the criteria for a maximal oxygen uptake were reached [22].

### Statistical procedures

Sample characteristics are presented as mean ± standard deviation for continuous variables and proportions for categorical variables. Pearson Chi-square test and independent samples t-test were used to assess potential sex differences. For BMI and weight, a non-parametric test (Mann-Whitney U) was conducted due to the lack of normal distribution. Data from the exercise logs are presented as proportions of the total number of exercise logs. Pearson Chi-square tests were run to assess the associations between frequency, intensity, type, location and social setting of exercise with sex and training group. The results were considered statistically significant if the *p*-value was less than 0.05. All statistical analyses were performed with SPSS 22 (Statistical Package for Social Science, Chicago, IL, USA).

## Results

The baseline characteristics of the study participants are presented in Table 1. No differences between the training groups existed at study entry. In both groups, men spent more hours in sedentary behavior and had significantly higher weight, height, and VO_2peak_ compared to women. Contrary, more women than men performed at least 30 min of daily physical activity (Table 1). The included participants had higher VO_2peak_ (11%) compared to those with no exercise logs. They also had higher VO_2peak_ (17%) and height (1.7%) compared to dropouts, but a lower BMI (3.7%) (*p* < 0.05). A higher proportion of the included participants performed 30 min of daily physical activity compared to the dropouts (77.3% vs. 66.1%, *p* < 0.05).

**Table 1:** Sample characteristics of the 618 study participants

	MCT			HIIT		
	All (*n* = 313)	Women (*n* = 152)	Men (*n* = 161)	All (*n* = 305)	Women (*n* = 139)	Men (*n* = 166)
Age (years)	72.3±1.9	72.2±1.8	72.5±2.0	72.4±2.0	72.5±1.9	72.2±2.0
Height (cm)	170.6±8.9	163.4±5.0	177.4±5.8*	170.6±8.9	163.2±5.2	176.9±6.0*
Weight (kg)	75.6±12.9	68.2±10.3	82.7±11.1*	75.6±14.0	66.5±9.9	83.3±12.3*
BMI (kg/m^2^)	25.9±3.6	25.6±3.8	26.3±3.4	25.8±3.5	25.0±3.5	26.6±3.3*
Sedentary time (h/d)	5.9±2.2	5.3±1.9	6.5±2.3*	5.6±2.1	5.0±1.7	6.0±2.3*
PA >30min/day (yes %)	77.8	83.3	72.5*	77.5	82.6	73.2*
VO_2peak_ (mL/kg/min)	29.3±6.6	26.4±5.1	32.0±6.7*	29.9±6.3	27.3±4.5	32.1±6.7*

Data are presented as mean ± standard deviation or proportions (%). *BMI* body mass index, *PA* physical activity, *VO*_*2peak*_ peak oxygen uptake

*Significantly different from women within the same training group (*p*<0.05)

### Frequency and intensity of exercise

**Fig. 2:**
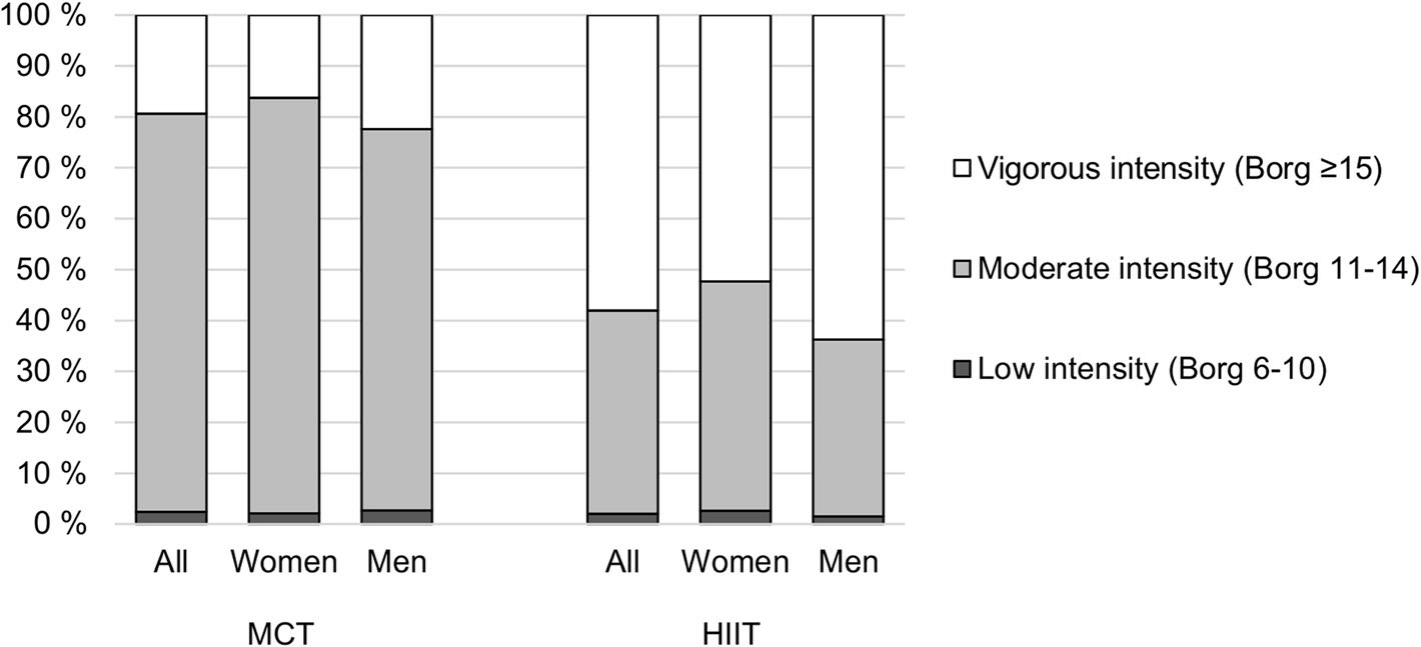
Exercise intensity. Data are presented as proportions of the total number of exercise sessions

The participants completed in total 69 492 exercise logs (33 608 HIIT group) during the year, of which 39 075 were received in prepaid envelopes and 30 417 in internet-based forms. Both groups performed 2.2 ± 1.3 exercise sessions per week. Almost 80% of the sessions in the MCT group were actually performed with moderate intensity (11–14 on the Borg scale), while almost 60% of the sessions in the HIIT group were performed with high intensity (≥15 on the Borg scale) (Fig. 2). In the MCT group, women had a significantly higher proportion of sessions with moderate intensity compared to men (81.7% vs. 74.9%, *p* < 0.01). In the HIIT group, men had a higher proportion of sessions with high intensity compared to women (63.7% vs. 52.3%, *p* < 0.01) (Fig. 2). In the MCT group, 9.6, 43 and 47.4% of the sessions had a duration of < 30 min, 30 min to 1 h, and more than 1 h, respectively. The corresponding percentages in the HIIT group were 10.1, 45 and 44.9%.

### Exercise type

**Fig. 3:**
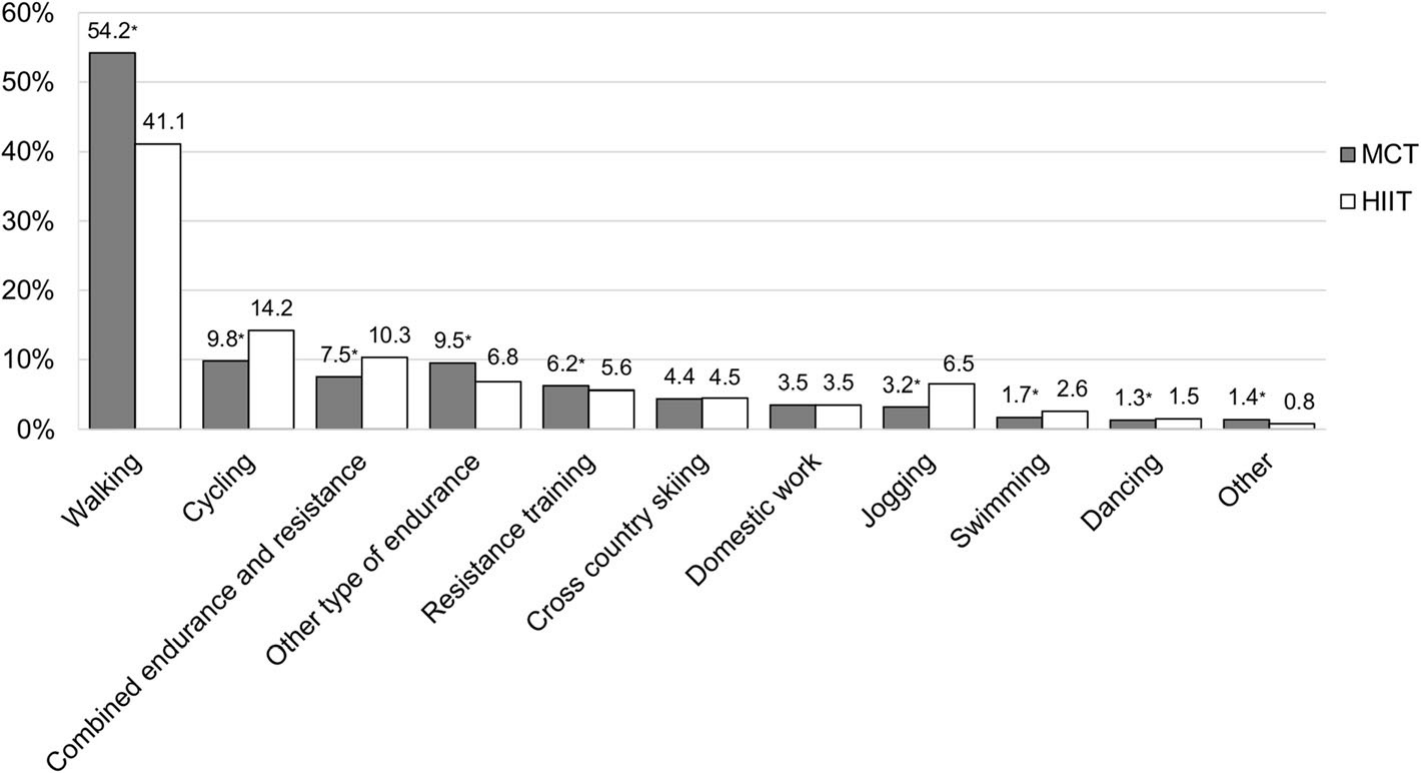
Association of exercise type with training group. Data are presented as proportions of the total number of exercise sessions. Other type of endurance; treadmill, cross trainer, aerobics etc., Domestic activities; housework, gardening etc., Other; golf, bowling, horseback riding etc. *Significantly different from the HIIT group (*p* < 0.05)

Walking was the most common exercise type in both training groups (Fig. 3). Compared to HIIT, MCT had a significantly higher proportion of sessions with walking and resistance training. Contrary, compared to MCT, HIIT had a higher proportion of sessions with cycling, combined endurance and resistance training, other types of endurance training (e.g. aerobic, treadmill), jogging, swimming and dancing. There were no group differences regarding cross-country skiing and domestic activities (e.g. housework, gardening) (Fig. 3).

**Fig. 4:**
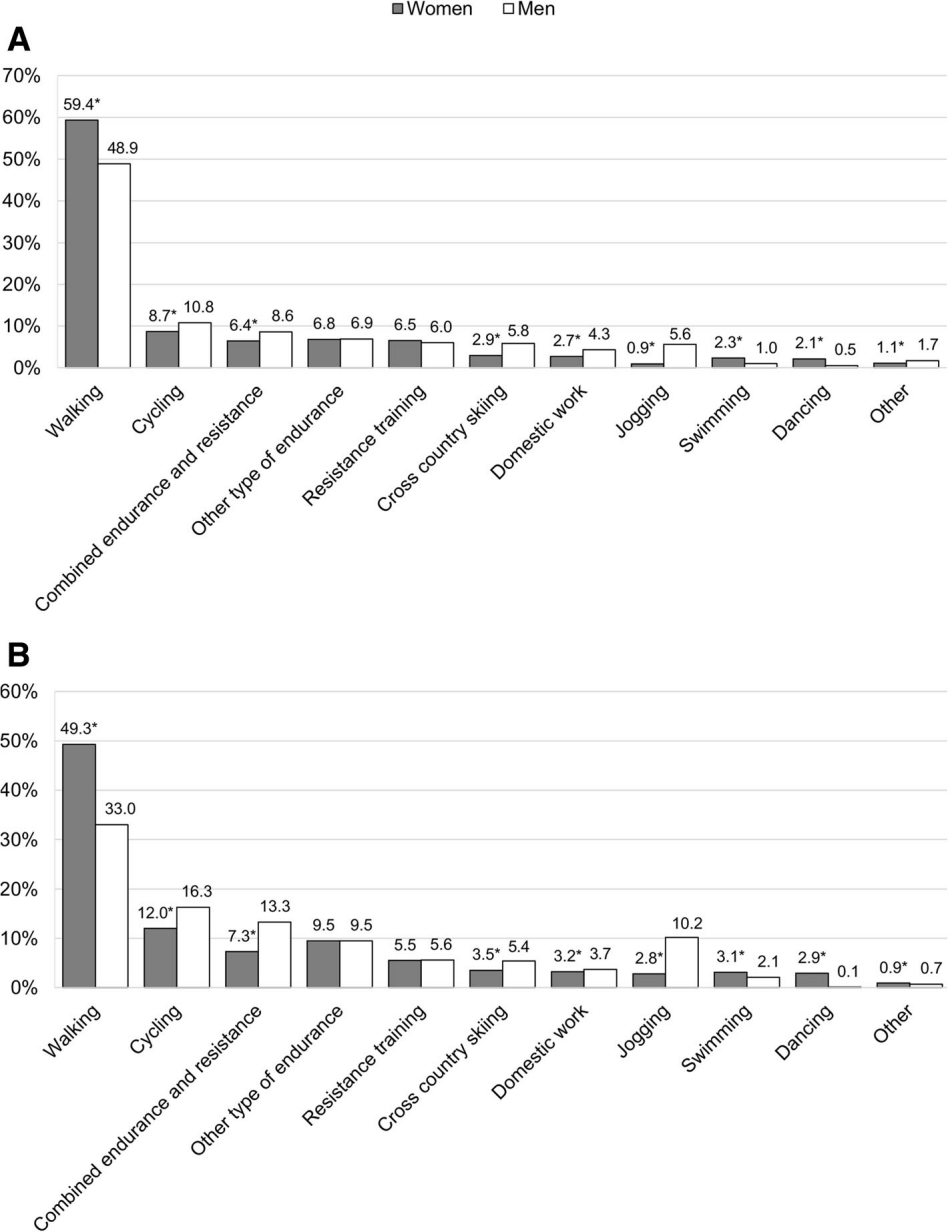
Association of exercise type with sex in the MCT (**a**) and HIIT (**b**) groups. Data are presented as proportions of the total number of exercise sessions. Other type of endurance; treadmill, cross trainer, aerobics etc., Domestic activities; housework, gardening etc., Other: golf, bowling, horseback riding etc. *Significantly different from men (*p* < 0.05)

In both groups, men had a higher proportion of cycling, cross-country skiing and jogging sessions compared to women (Fig. 4). Men also had a higher proportion of sessions with combined endurance and resistance training and domestic activities than women. In contrast, women had a higher proportion of walking, swimming and dancing sessions than men. There were no sex differences in resistance training and other types of endurance training (Fig. 4).

### Location of exercise

**Fig. 5:**
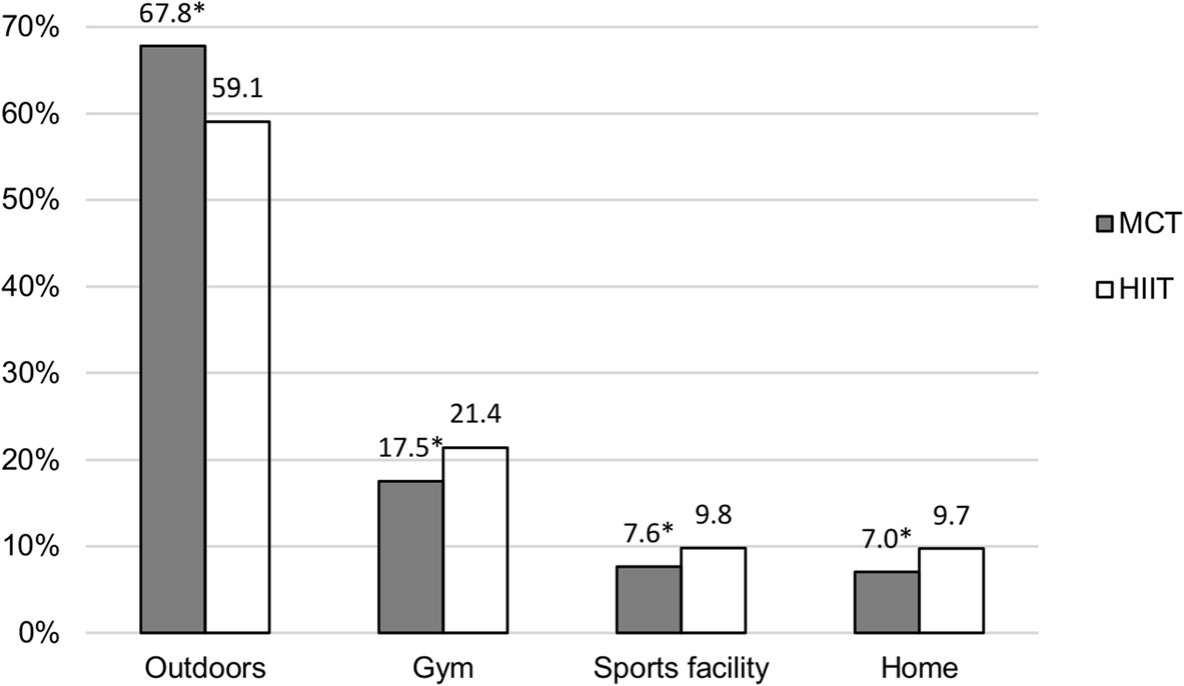
Association of exercise location with training group. Data are presented as proportions of the total number of exercise sessions. *Significantly different from the HIIT group (*p* < 0.05)

Both groups exercised most frequently outdoors in nearby area and in nature (Fig. 5). Additional analyses showed that outdoors was the most frequently reported exercise location in both warmer (April–October) and colder (November–March) months. The MCT group had a significantly higher proportion of sessions outdoors than the HIIT group. Contrary, compared to the MCT group, HIIT had a higher proportion of sessions at a gym, sports facility and at home (Fig. 5).

**Fig. 6:**
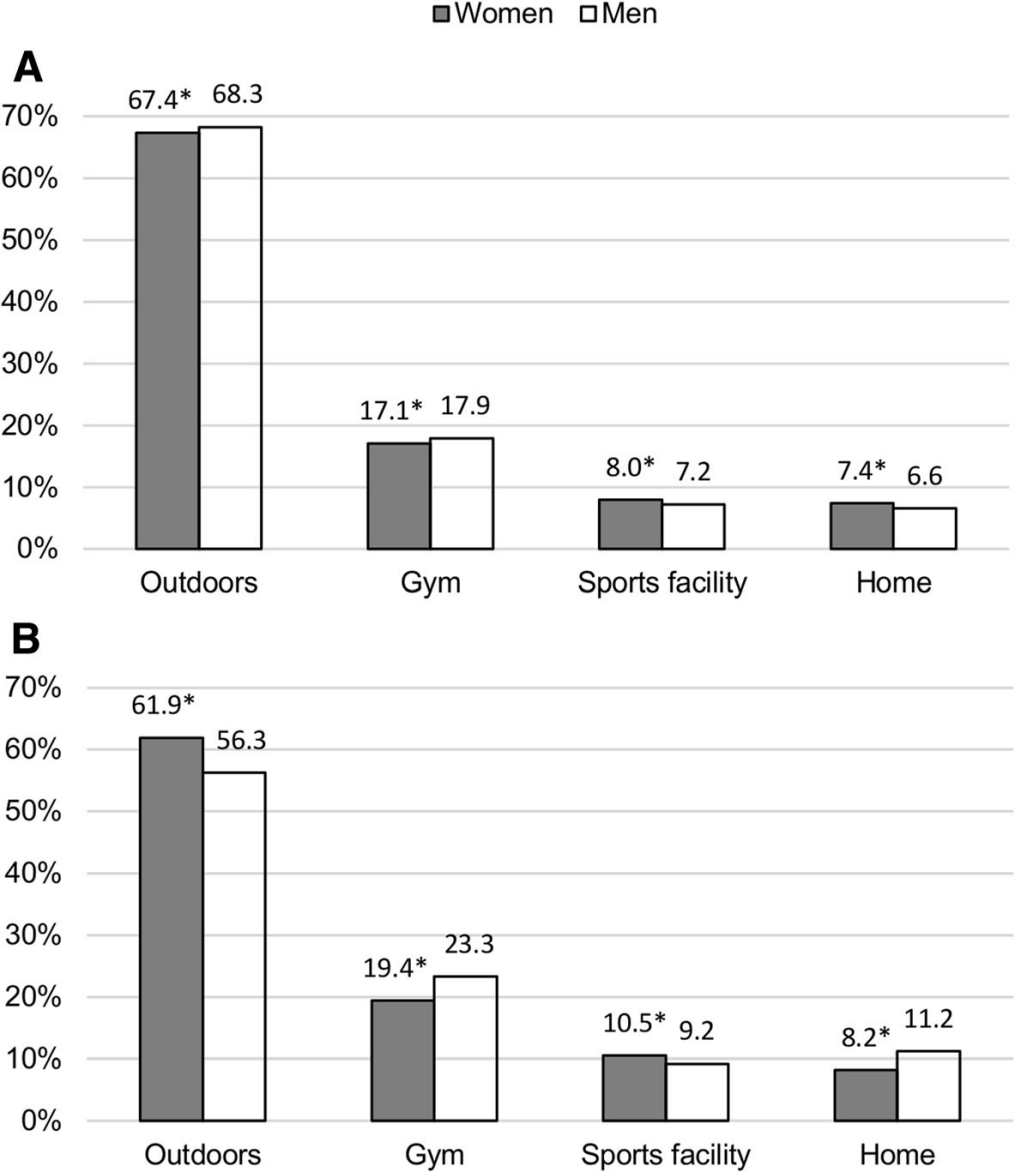
Association of exercise location with sex in the MCT (**a**) and HIIT (**b**) groups. Data are presented as proportions of the total number of exercise sessions. *Significantly different from men (*p* < 0.05)

In both groups, men had a significantly higher proportion of sessions at a gym compared to women (Fig. 6). Contrary, women had a higher proportion of sessions at a sports facility compared to men. In the MCT group, men had a significantly higher proportion of sessions outdoors compared to women, while the opposite was observed in the HIIT group (Fig. 6).

### Social setting of exercise

Both groups performed an equal proportion of exercise sessions alone (MCT: 50%, HIIT: 49.6%) and together with others (MCT: 50%, HIIT: 50.4%). In both groups, women had a significantly higher proportion of sessions together with others compared to men (56% vs. 44%, *p* < 0.01). The HIIT group had a significantly higher proportion of sessions organized by Generation 100 compared to the MCT group (8.1% vs. 5.9%, *p* < 0.01).

## Discussion

This is the first study that has followed older adults instructed to perform MCT or HIIT over a one-year period, collected data from each exercise session they performed and provided descriptive data on their exercise patterns. The main finding is that both groups to a large degree exercised with the prescribed intensity. MCT had a higher proportion of walking sessions than HIIT, while HIIT had a higher proportion of jogging sessions than MCT. In addition, HIIT had a higher proportion of sessions with cycling, combined endurance and resistance training, swimming and dancing. Both groups exercised more frequently outdoors than indoors and performed an equal amount of sessions alone and together with others.

### Frequency and intensity of exercise

Evidence from HIIT studies conducted under controlled laboratory conditions has provided proof-of-concept of efficacy [9]. However, it has been argued that HIIT has high efficacy but low effectiveness [16], and long-term exercise interventions carried out under free-living conditions have been asked for to investigate whether HIIT is feasible as a public health initiative among older adults [9, 16]. Our data showed that both training groups reported on average more than two exercise sessions per week throughout the year. Approximately 60% of the sessions in the HIIT group were performed with a self-reported high-intensity (≥15 Borg scale), indicating that older adults are able to perform HIIT over a long time-period without strict supervision. However, women had a lower proportion of sessions with high-intensity exercise compared to men. This result is in line with previous findings that women (aged 60–67 years) are less likely than men to prefer vigorous physical activity [23].

### Exercise type

In line with the previous literature, our study showed that walking was the most common exercise type among older adults [24, 25]. This result is not surprising as walking is among the most cost effective and accessible means of exercise [26]. In addition, walking has been identified as a relatively safe exercise alternative to older adults [25]. We found that walking was the most common exercise type in both training groups. However, the MCT group had a higher proportion of walking sessions than the HIIT group, while the HIIT group had a higher proportion of sessions with for instance jogging and cycling. This might indicate that some older adults in the HIIT group feel that it is easier to achieve a high-intensity level when performing jogging and cycling compared to walking. Absolute workload at a given intensity varies greatly among individuals with different levels of cardiorespiratory fitness (CRF) [27], so that e.g. walking at 5 km/h corresponds to moderate intensity for an individual with relatively high CRF level, while the same speed exhibits near-maximal intensity for an individual with low CRF. Therefore, the type of exercise an individual need to perform in order to achieve a feeling of high intensity varies from one individual to another [27]. Since ageing often results in CRF decline [28], it is likely that many older adults will reach a feeling of high-intensity when walking. However, those with a high CRF level might need to perform other exercise types, for instance jogging and cycling, to reach the same intensity level during their workout session.

In line with Martin and colleagues [29] we found that women more often engaged in walking, swimming and dancing compared to men, while men more often performed jogging, cycling and winter sports. Our data also showed that men performed a higher amount of sessions with domestic activities and combined endurance and resistance training compared to women. The sex differences were the same in both training groups, indicating that disparities in type of exercise between older women and men are independent of the exercise intensity they are instructed to perform.

### Location of exercise

Outdoors in nearby area and in nature was the most frequently reported exercise location in both training groups. This finding is in line with previous studies reporting that older adults prefer to exercise close to home [23, 30] and outdoors [23]. Interestingly, outdoors was the most common exercise location in both warmer and colder months despite the fact that colder months in Norway consist of more snow, higher prevalence of ice and relatively fewer hours of daylight compared to warmer months. The HIIT group had a higher proportion of sessions at a gym and sport facility compared to the MCT group. This finding is likely related to the fact that the HIIT group reported a higher proportion of sessions with exercise types commonly performed at these locations (e.g. swimming and other types of endurance training) compared to the MCT group. Some older adults might feel that it is easier to reach a high-intensity level with activities located at a gym and sports facility compared to outdoors.

### Social setting of exercise

Our results showed that both the MCT and HIIT group performed an equal amount of exercise sessions alone and together with others, suggesting that both individual and group-related exercise intervention strategies may be attractive to older adults. However, women exercised more frequently together with others than men. This result is in line with previous findings that women aged 60–67 years are less likely than men to prefer physical activity that can be done alone [23], and that more women than men express a need for social support to maintain an exercise program [31].

Altogether, our findings showed that older adults engage in a variety of exercise types, especially when instructed to perform HIIT, suggesting that future exercise interventions might profit of giving older adults the choice of different exercise types instead of offering only one. Our findings also suggest that interventions to promote exercise in older adults should focus on both indoor and outdoor environments. The popularity of exercising outdoors in both colder and warmer months highlight the importance of facilitating outdoors areas such as hiking trails. Furthermore, our findings show that sex differences in exercise patterns exist and need to be taken into consideration when designing exercise programs targeting older men and women. Given the increasing number of older adults [1] and the health benefits associated with exercise [32], information on how to get older adults to exercise and maintain their exercise behavior is important. The results of the present study can help clinicians and researchers to develop exercise programs targeting older adult’s interests and in that way improve long-term participation.

### Strengths and limitations

The main strength of this study is the large data material on exercise patterns. Most research on exercise pattern has used a cross-sectional design whereas we followed older adults over a one-year period and collected data from each exercise session they performed. Furthermore, this is the first study to assess differences in exercise patterns between older adults instructed to follow MCT versus HIIT.

Since our data is self-reported, we do not know for sure if we have data from all exercise sessions performed throughout the year. Furthermore, subjective measures are susceptible to recall bias, especially among older adults [17, 18]. However, our results are based on nearly 70000 exercise logs, which is the largest data material on exercise patterns in older adults. In addition, exercise logs have an advantage over the widely employed exercise questionnaires where the subject is asked to recall exercise performed in the past as opposed to recording the exercise right after the moment of occurrence, as is the case with exercise logs.

Selection bias may limit generalizability to other populations of older adults since the included participants in the Generation 100 study were healthier, more educated and more physically active than nonparticipants [19]. However, our study population was diverse and included both healthy as well as older adults with comorbidities, and both inactive and very active older adults were included. The findings in the present study are based on a very large data material, and represent the most comprehensive data material on exercise patterns among older adults to date.

## Conclusions

Our findings show that older adults are able to perform both MCT and HIIT without strict supervision. Furthermore, older adults randomized to MCT versus HIIT have different patterns of exercise type and location of exercise, while there are no differences in social setting of exercise. The observed sex differences were the same in both training groups. Clinicians and researchers might capitalize on our findings when planning future exercise interventions targeting older adults. Our findings may also provide important information for future public health initiatives in order to provide tailored exercise recommendations.

## Abbreviations

BMI: Body mass index VO_2peak_ peak oxygen uptake
CRF: Cardiorespiratory fitness.
HIIT: High-intensity interval training
MCT: Continuous moderate-intensity training
RPE: Rating of perceived exertion
